# Functional networks are impaired by elevated tau-protein but reversible in a regulatable Alzheimer’s disease mouse model

**DOI:** 10.1186/s13024-019-0316-6

**Published:** 2019-03-27

**Authors:** Claudia Green, Astrid Sydow, Stefanie Vogel, Marta Anglada-Huguet, Dirk Wiedermann, Eckhard Mandelkow, Eva-Maria Mandelkow, Mathias Hoehn

**Affiliations:** 1 In-vivo-NMR Laboratory, Max Planck Institute for Metabolism Research, Gleuelerstrasse 50, D-50931 Cologne, Germany; 2 Max-Planck-Institute for Metabolism Research, Hamburg Outstation, c/o DESY, Notkestrasse 85, 22607 Hamburg, Germany; 3 German Center for Neurodegenerative Diseases (DZNE), Ludwig-Erhard-Allee 2, 53175 Bonn, Germany; 4 CAESAR Research Center, Ludwig-Erhard-Allee 2, 53175 Bonn, Germany; 5 Department of Radiology, Leiden University Medical Center, Leiden, The Netherlands; 6 Percuros B.V., Enschede, The Netherlands

**Keywords:** Resting state-fMRI, Alzheimer’s disease, Tau, Soluble tau, Transgenic mouse models, Diffusion MRI, Functional connectivity, Functional networks

## Abstract

**Background:**

Aggregation of tau proteins is a distinct hallmark of tauopathies and has been a focus of research and clinical trials for Alzheimer’s Disease. Recent reports have pointed towards a toxic effect of soluble or oligomeric tau in the spreading of tau pathology in Alzheimer’s disease. Here we investigated the effects of expressing human tau repeat domain (tauRD) with pro- or anti-aggregant mutations in regulatable transgenic mouse models of Alzheimer’s Disease on the functional neuronal networks and the structural connectivity strength.

**Methods:**

Pro-aggregant and anti-aggregant mice were studied when their mutant tauRD was switched on for 12 months to reach the stage where pro-aggregant mice show cognitive impairment, whereas anti-aggregant mice remained cognitively normal. Then, mutant tauRD was switched off by doxycycline treatment for 8 weeks so that soluble transgenic tau disappeared and cognition recovered in the pro-aggregant mice, although some aggregates remained. At these two time points, at baseline after 12 months of mutant tau expression and after 8 weeks of doxycycline treatment, resting state fMRI and diffusion MRI were used to determine functional neuronal networks and fiber connectivities. Results of the transgenic mice were compared with wildtype littermates.

**Results:**

Functional connectivity was strongly reduced in transgenic animals during mutant tauRD expression, in relation to WT mice. Interestingly, transgenic mice with the non-aggregant tau mutant showed identical functional deficits as the pro-aggregant mice, even though in this case there was no cognitive decline by behavioral testing. Upon 8 weeks doxycycline treatment and transgene switch-off, functional connectivity in both transgenic groups presented complete normalization of functional connectivity strength, equivalent to the situation in WT littermates. Structural connectivity was found only marginally sensitive to mutant tau expression (both pro- and anti-aggregant tauRD) and by doxycycline treatment.

**Conclusions:**

Our in vivo investigations unravel for the first time a strong reduction of functional neuronal networks by the presence of increased soluble rather than fibrillary tau, independent of its intrinsic propensity of aggregation, which is reversible by switching tau off. Our functional MRI study thus is an unexpected in vivo validation of a novel property of tau, while previous results pointed to a role of aggregation propensity for a pathological state by histopathology and cognitive decline. Our results present further evidence for early tauopathy biomarkers or a potential early stage drug target by functional networks analysis.

**Electronic supplementary material:**

The online version of this article (10.1186/s13024-019-0316-6) contains supplementary material, which is available to authorized users.

## Background

Most neurodegenerative diseases which are associated with cognitive decline, such as Alzheimer’s Disease (AD), Progressive Supranuclear Palsy (PSP) and Frontotemporal Dementia (FTD) are accompanied by abnormal hyperphosphorylation and aggregation of tau protein [1, 2]. Tau is a microtubule-associated protein located mostly in the axons of neurons. Under normal conditions it stabilizes microtubules, thereby supporting axonal extension and intracellular transport by motor proteins. In tauopathies, however, tau becomes abnormally phosphorylated at multiple sites, assembles in the form of paired helical filaments **(**PHF**)** and eventually aggregates to insoluble neurofibrillary tangles (NFT) inside neurons [3]. The deposition and accumulation of aggregated tau represents a pathological marker of AD [4, 5] which progresses stereotypically in the brain and leads to microtubule destabilization, decay of synapses, and finally neuronal cell death. Despite considerable efforts, the role of tau in AD progression and neurotoxicity is not yet fully understood. Recent investigations point towards a strong correlation of neurotoxicity with the soluble oligomeric forms of tau rather than with the accumulation of NFT [6–9].

Magnetic Resonance Imaging (MRI) enables to non-invasively monitor cerebral changes on a microstructural scale in vivo and repetitively. Diffusion-sensitized MRI (dMRI) maps microstructural diffusion processes by contrasting the tissue-dependent intra- and extracellular water diffusion. dMRI is nowadays irreplaceably used in the preclinical and clinical environment to monitor neurological abnormalities qualitatively, as well as quantitatively by calculating diffusion parameter maps, such as the anisotropy values. Advanced dMRI methods such as Q-ball imaging (QBI) [10] are even capable of reconstructing and visualizing structural neuronal pathways, so-called fiber tracts that allow a three-dimensional visualization of the white matter microstructural architecture, and have become an established key method to compute cerebral structural connectivity.

A prominent approach to analyze functional brain connectivity without specific stimulus, i.e. in a resting brain condition, is resting-state functional MRI (rs-fMRI) [11, 12]. rs-fMRI is based on correlating the time courses of blood-oxygen-level-dependent (BOLD) [13] MRI signal changes between single voxels or within an entire region of interest (ROI) and has been proven to reliably reflect neural activity under resting conditions [14, 15]. Several groups of brain regions that show a high temporal correlation with each other through this method are considered to be functionally linked and further considered as a functional network.

Diffusion MRI and resting-state functional MRI techniques now serve as the dominant methods to study structural and functional connectivity of the brain and have generated new significant results in the understanding of such complex diseases such as AD. Several clinical studies provided evidence for MRI connectivity measures to be used as an early biomarker for AD onset and progression [16–18]. The availability of transgenic mouse models allows studying pathological characteristics under standardized conditions over the span of a lifetime. In recent years, preclinical research in AD largely focused on characterizing the impact of amyloidosis, one of the currently considered major driving events of AD [19–22]. The role of tau in the complex interplay of this pathology has been mostly limited to ex vivo and biochemical analysis. Only few preclinical, longitudinal studies using MRI in vivo focused on the description of the structural consequence of continuous tau progression and accumulation [23–25].

Here we determined the whole brain connectivity in transgenic mouse models of tauopathy with regulatable human tau expression. As transgenes we used the human tau repeat domain (tauRD, responsible for tau aggregation) with two types of point mutations, rendering them either pro-aggregant or anti-aggregant [26]. In these models, we aimed to investigate the effect of switching-off tau expression on the functional brain network. Previous histopathological investigations of these mouse models have shown the relation between tau expression, tau aggregate accumulation and memory decline [27, 28]. Upon shutting down human tau expression, recovery of memory functions was observed by behavioral tests, along with the gradual disappearance of human tau aggregates [29]. Here, we analyzed the intrinsic structural and functional connectivity and their network properties before and after reversing tau expression at a late stage of pathological development. By the combined use of dMRI and rs-fMRI, we further evaluated under controlled experimental conditions the benefit of tau as a therapeutic clinical target [30].

## Materials and methods

### Animals

All animal experiments were carried out in accordance with the guidelines of the German Animal Welfare Act and approved by the local authorities (Landesamt für Natur, Umwelt und Verbraucherschutz Nordrhein-Westfalen) under the animal permission 84–02.04.2014.A369. Animals were socially housed under a fixed 12:12 h light/darkness cycle with ad libitum access to food and water. In total 23 female mice of 10–12 months old age, provided by the Mandelkow lab, were used in this study on functional connectivity. Generation, characterization, histopathological and behavioral analysis of the mice were already described in detail in [27, 28]. The cohort consisted of two transgenic mouse strains that express mutant variants of the truncated tau four-repeat domain (TauRD) [27] (pro-aggregant *n* = 8 / anti-aggregant n = 8) and non-transgenic littermates (*n* = 7). Tau expression in the pro-aggregant mice (TauRD/ΔK280) leads to the strong formation of tau aggregates with subsequent decay of synapses and neurons, primarily in the hippocampal regions [28, 29] and signs of cognitive impairment [31]. In the anti-aggregant mouse line, two proline mutations (TauRD/ΔK280/PP) were additionally inserted into the truncated human tau, to disable tau aggregation with continuing expression [32]. These latter mice do not develop pathological changes in the brain, no loss of spines or synapses, and no cognitive decline. They served as a disease-resistant comparison. The non-transgenic littermates served as controls. The expression of tau was suppressed via the administration of doxycycline (by the Tet-Off gene expression system) [33, 34]. An additional 20 animals (pro-aggregant *n* = 8, and anti-aggregant n = 8 transgenic mice and wildtype litter mates *n* = 4) were used for confirmation of the ON-state (n = 4) and OFF-state (n = 4), respectively, of the transgene by Western blot analysis.

### Experimental protocol

Two structural and functional MRI datasets were acquired over the time course of 2 months. At the first time point, the animals were 10–12 months old. This measurement served as a baseline of the situation with fully expressed human tau in both transgenic groups, the pro-aggregant and the anti-aggregant mice (transgenic tau ON-state). There, the pathological condition was fully developed in the pro-aggregant mice. After this first measurement, human tau expression was continuously suppressed by administering doxycycline-containing food pellets (200 mg Doxycycline/kg - ssniff Spezialdiäten GmbH, Soest, Germany) to all (transgenic and wild type) animals for the remaining study period. The second MRI measurement was performed after eight weeks of continuous doxycycline uptake. The transgenic human tau is completely switched off (transgenic tau OFF state) within 2–3 days of doxycycline administration and disappears below detectability within 4 weeks [27]. At the end of the experimental in vivo protocol, all animals were transcardially perfused for further biochemical and histological validations. In particular, the successful suppression of human tau by the 8 weeks of doxycycline application was confirmed by Western blotting in Additional file 1: Figure S1, in full agreement with earlier characterization [27].

### MRI data acquisition

MRI datasets were generated on a small animal 9.4 T horizontal MRI system with a 20 cm bore diameter, actively shielded BGA12S2 gradient coils, and a transmit/receive 1H quadrature cryogenic surface coil (Bruker BioSpin, Ettlingen, Germany). Image acquisition was executed with ParaVision 5.1 software (Bruker BioSpin GmbH). The animal’s body temperature was measured via a fiber optic rectal probe (SA Instruments, NY, USA) and kept constant at 37 °C ± 1.0 °C by an adjustable water circulating system (medres, Cologne, Germany). Breathing rate and body temperature were continuously monitored (1025 T System, SA Instruments, NY, USA) and recorded (DASYlab Software, Measurement Computing, Norton, USA). Animals were initially anesthetized with 2% Isoflurane in a mixture of 70/30% N_2_/O_2_ continuous gas flow for stable positioning. Heads were fixed in a dedicated animal cradle including tooth and ear bars to minimize movement artifacts throughout the scan session; subsequently Isoflurane was reduced to 1.5% at the beginning of the scanning session. Diffusion-sensitized structural MRI and BOLD rs-fMRI were acquired for each time point within one session to allow for good temporal correlation. At the beginning of each MRI session, a FieldMap with consecutive local shim was run to improve magnetic field homogeneity, followed by a T2-weighted TurboRARE sequence as an anatomical reference scan with a field of view (FOV) of 17.5 × 17.5 mm^2^, 48 contiguous slices of 0.2 mm slice thickness, matrix dimension of 256 × 256, repetition time (TR) = 5500 ms, echo time (TE) = 32.5 ms, and a RARE factor of 8 with two averages. Meanwhile, a bolus of 0.1 mg/kg Medetomidine (Domitor®, Elanco), suspended in 250 μl NaCl, was injected subcutaneously and Isoflurane level was reduced, according to the breathing rate, down to 0.5%, following a protocol described earlier [22, 35]. 15–20 min waiting time between Medetomidine bolus and rs-fMRI scan was kept to ensure successful switching of anesthesia.

Functional MRI was then acquired with a gradient-echo echo-planar imaging (GE-EPI) sequence with 105 repetitions, FOV: 17.5 × 17.5 mm^2^, matrix size: 96 × 96, in-plane resolution: 182 × 182 μm^2^, TR/TE = 2840 ms/18 ms, and 16 slices of slice thickness 0.5 mm.

Diffusion-sensitized MRI was conducted with a half-sphere Q-ball imaging protocol of 126 diffusion encoding gradient directions of half a unit sphere in a 4-shot spin-echo EPI sequence (SE-EPI). A constant b-value was set to 2000 s/mm^2^ [36], ∆/δ = 10 ms/4 ms, FOV: 17.8 × 17.8 mm^2^, matrix size: 128 × 128, leading to an in-plane resolution of 139 × 139 μm^2^; TR/TE = 3500 ms/20 ms. 22 contiguous slices with a slice thickness of 0.5 mm covered the entire forebrain and parts of the cerebellum and olfactory bulb. All diffusion directions were split evenly in 8 separate scans to allow inclusion of one non-diffusion encoded image (b = 0 s/mm^2^) linearly distributed between the diffusion sensitized images, preceding each scan.

### MRI data processing

For each diffusion and functional dataset, individually coregistered mouse brain atlases of the hippocampus [37], the neocortex [38], and the basal ganglia [39], were generated, which map onto the diffusion and functional images to allow automatic multiple Regions of Interest (ROI) extraction, as described in detail in [35]. Besides the hippocampus (Hp), major cortical (M1/M2 (motor), S1, S2 (somatosensory), VC (visual), AC (auditory), MO (medial orbital), EntC (entorhinal), PrL (prelimbic), RSD/RSG (retrosplenial dysgranular/granular), Cg (cingulate)) and subcortical (GP (globus pallidus), CPu (caudate putamen), Th (thalamus), HyTh (hypothalamus)) ROIs were chosen for hemisphere dependent and independent data analysis. These regions are associated with memory formation, sensorimotor processing or part of the default mode network (DMN) [40–43]. To account for the structural transport of neuronal signals between the above-mentioned regions of interest and to meet the purpose of diffusion imaging, seven major white matter tracts were exclusively included in the analysis of the diffusion datasets, namely corpus callosum (cc), internal capsule (ic), fornix (fx), fimbria (fi), amygdala (amy), cerebral peduncle (cp) and anterior commissure (ac).

The functional datasets were brain extracted, slice-wise motion corrected (FMRIB Software Library; http://www.fmrib.ox.ac.uk/fsl [44, 45]), linearly detrended [46] and physiological noise (recorded respiratory signals, motion parameters, and drifts up to the second order) was regressed out [46]. In addition, we applied in-plane spatial smoothing with a Gaussian filter of FWHM = 0.3 mm (FSL SUSAN), a bandpass-filter of bandwidth 0.01–0.08 Hz, and finally the data were normalized.

To evaluate temporal within and between group-effects, the ROI-average time series of each dataset was computed, and between each pair of ROIs (nodes of the network) the full Pearson correlation coefficient was obtained and the Fisher z-values were group-wise averaged using a customized version of FSLNets (v0.6; www.fmrib.ox.ac.uk/fsl). Additionally, the strength of the individual ROI was analyzed: all voxels within a region of interest were correlated and the results group-wise averaged using custom-written scripts after Fisher’s z transformation.

A multi-step process was carried out for motion correction of the diffusion datasets. First, brain extraction and motion correction were performed on the A0 images only (FMRIB Software Library; http://www.fmrib.ox.ac.uk/fsl [44, 45]). The obtained matrices were consecutively interpolated and lastly applied to the corresponding diffusion images by an in-house script. ROIs were extracted using the A0 images, following the steps already described above for the functional datasets. Spherical harmonics-based Q-ball reconstruction [47, 48] was achieved using DSIStudio (dsi-studio.labsolver.org) with a regularization parameter of 0.006 [47] and 8-fold tessellation. We performed deterministic streamline whole-brain tractography [49] on the reconstructed datasets with DSIStudio and the following tracking criteria: QA termination threshold of 0.03, 0.5 mm step size, 0.1 weighting, a fiber length constraint of 5–120 mm and a maximal turning angle of 55°. Fiber density was calculated by counting the fibers that pass through or end in each pair of ROI (DSIStudio). The result was voxel-normalized, group-averaged and connections with less than one fiber per voxel were set to zero value with MATLAB v.2014b (The MathWorks, Inc., Natick, Massachusetts, United States).

### Statistical analysis

Statistical analysis was performed with MATLAB v.2014b (The MathWorks Inc., Natick, MA, USA) and GraphPad Prism v. 7.00, (GraphPad Software, La Jolla California USA). The matrix datasets did not survive the assumption of normality tested with the Shapiro-Wilk normality test. A Friedman test was run for treatment dependent effects within each group, a Kruskal-Wallis analysis of variance by ranks with Dunn’s post hoc correction for multiple comparison for the between group effects within each time point. Statistical significance levels were set to *p* < 0.05:*, *p* < 0.005:**. All *p*-values given in this study are adjusted values based on multiple comparisons.

## Results

### Functional connectivity

**Fig. 1:**
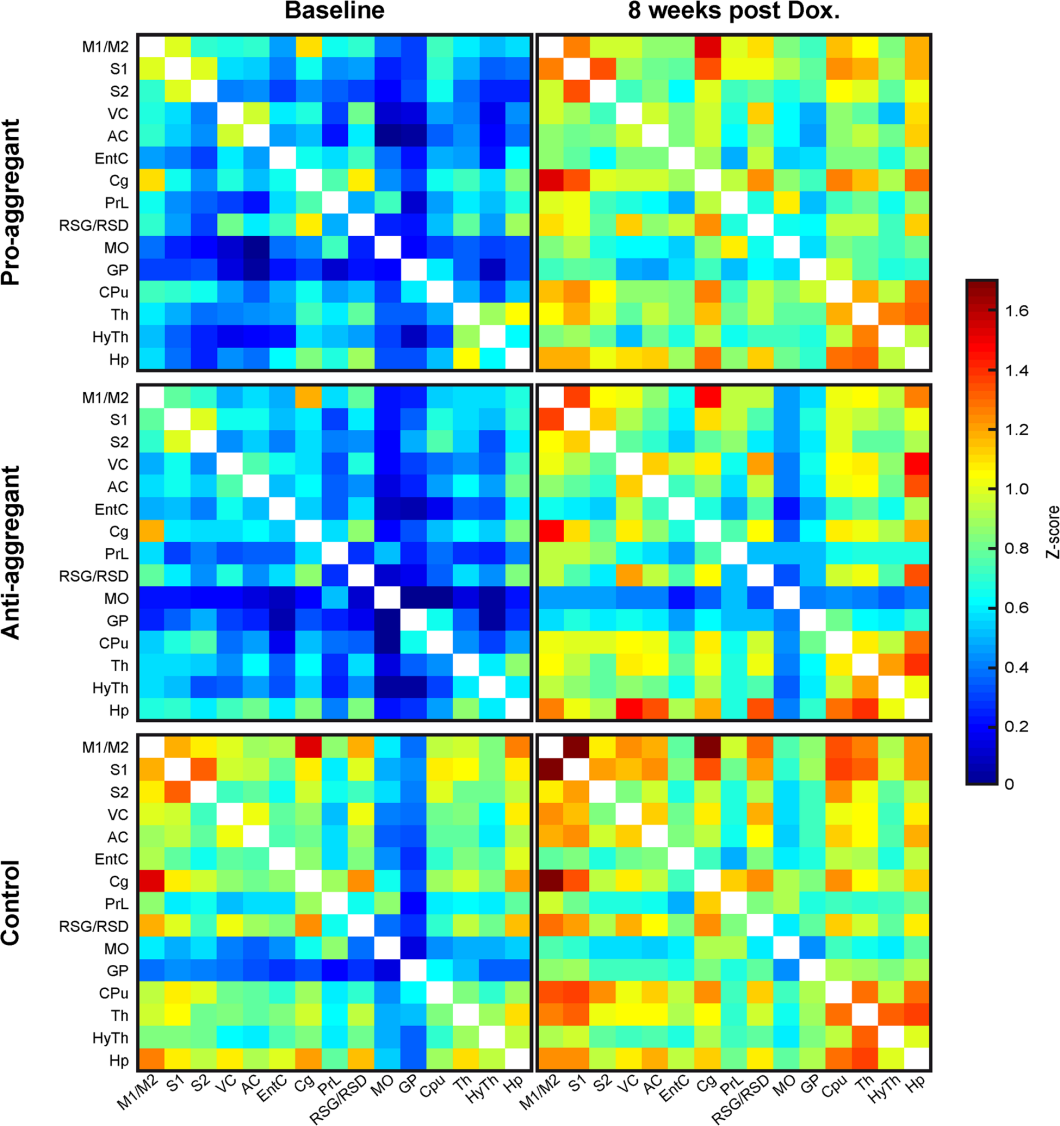
Functional connectivity matrices of transgenic and wildtype mice during baseline and after 8 weeks treatment with doxycycline. The pro- and anti-aggregant mice show matrices of clearly lower z-score values at baseline condition (left column). Upon 8 weeks of doxycycline treatment (right column), the z-score values of the transgenic animals closely approximate those of the control group

The baseline (pre-treatment) measurement represents the fully developed expression of the exogenous human tau repeat domain for the two transgenic groups, with strong tau pathology (tau aggregation, synaptic decay, neuronal loss, memory deficits) in the pro-aggregant transgenic animals, but no pathology in the anti-aggregant animals. At this time point, the functional connectivity matrices comprised of memory associated regions, the sensorimotor network and the default mode network of the pro- and anti-aggregant transgenic mice are compared with the matrix of the control group, consisting of wildtype littermates (Fig. 1, left column). The matrices of both transgenic animal groups show clearly lowered connectivity strength across the whole networks relative to the control group, as visualized by the overall color change to blue, indicating substantially lower z-score values in the LUT. Both, the pro-aggregant and the anti-aggregant tau transgenic groups present almost identical functional connectivity strengths across all networks at baseline conditions when the transgene human tau is fully expressed.

After eight weeks of treatment with doxycycline, given through the food pellets, expression of human tau in both soluble and aggregated forms, is completely reduced (Additional file 1: Figure S1), as already demonstrated earlier [28]. At this time, the functional connectivity matrices of the two transgenic groups show clearly enhanced values (Fig. 1, right column) compared to the baseline measurement, closely approximating the overall functional connectivity strength of the control group of WT littermates at baseline. This strengthening of the functional network by doxycycline-induced tauRD switch-off is even statistically significant for several correlations as demonstrated in the *p*-value matrices, generated from the consecutive z-score matrices within each group (Additional file 2: Figure S2). This is particularly pronounced for connectivity of the hippocampus and thalamus with all sensorimotor cortical regions in the pro-aggregant animals. At the same time, the non-transgenic littermate control group, having been fed the same doxycycline containing food pellets, also presented a slight increase of z-score values in the functional connectivity matrix (Fig. 1, right column). However, this was not significant, except for the two connections of cingulate cortex with prelimbic cortex (*p* = 0.0498) and of visual cortex with globus pallidus (*p* = 0.0498).

**Fig. 2:**
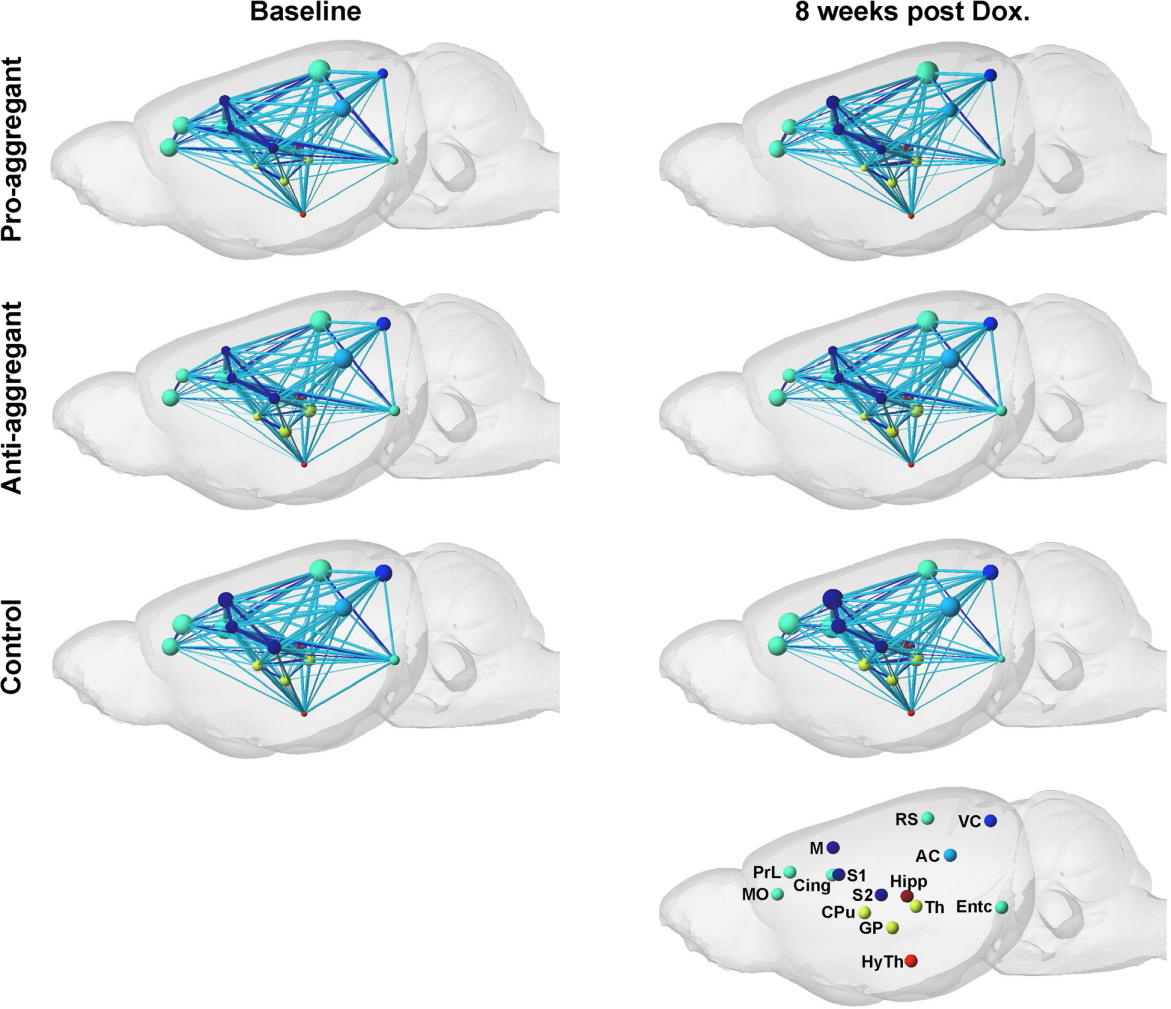
Functional network changes. In this schematic the functional connectivity between pairs of nodes is marked by lines of increasing thickness with increasing z-score values, indicating increasing functional connectivity. The intra-node strength is depicted by larger or smaller spheres

Additional analysis of the intra-node connectivity, reflecting the functional strength within each ROI itself allowed the separation between changes of the functional connectivity (FC) strength *within* individual nodes and the FC strength *between* nodes. The intra-node strength is schematically depicted by spheres with differing sizes while the inter-node FC strength is presented by differing line widths (Fig. 2). Larger spheres and line widths represent stronger connectivity strength. Prominent changes of the intra-node strength are not found between the baseline and the treatment time point, indicating that the doxycycline treatment leads predominantly to strengthening of the inter-node connectivity strengths in the transgenic animals. Concurrently, the intra-node strengths of regions in the somatosensory cortex are persistently lower for the pro- and anti-aggregant mouse models than for the control group of wildtype mice.

**Fig. 3:**
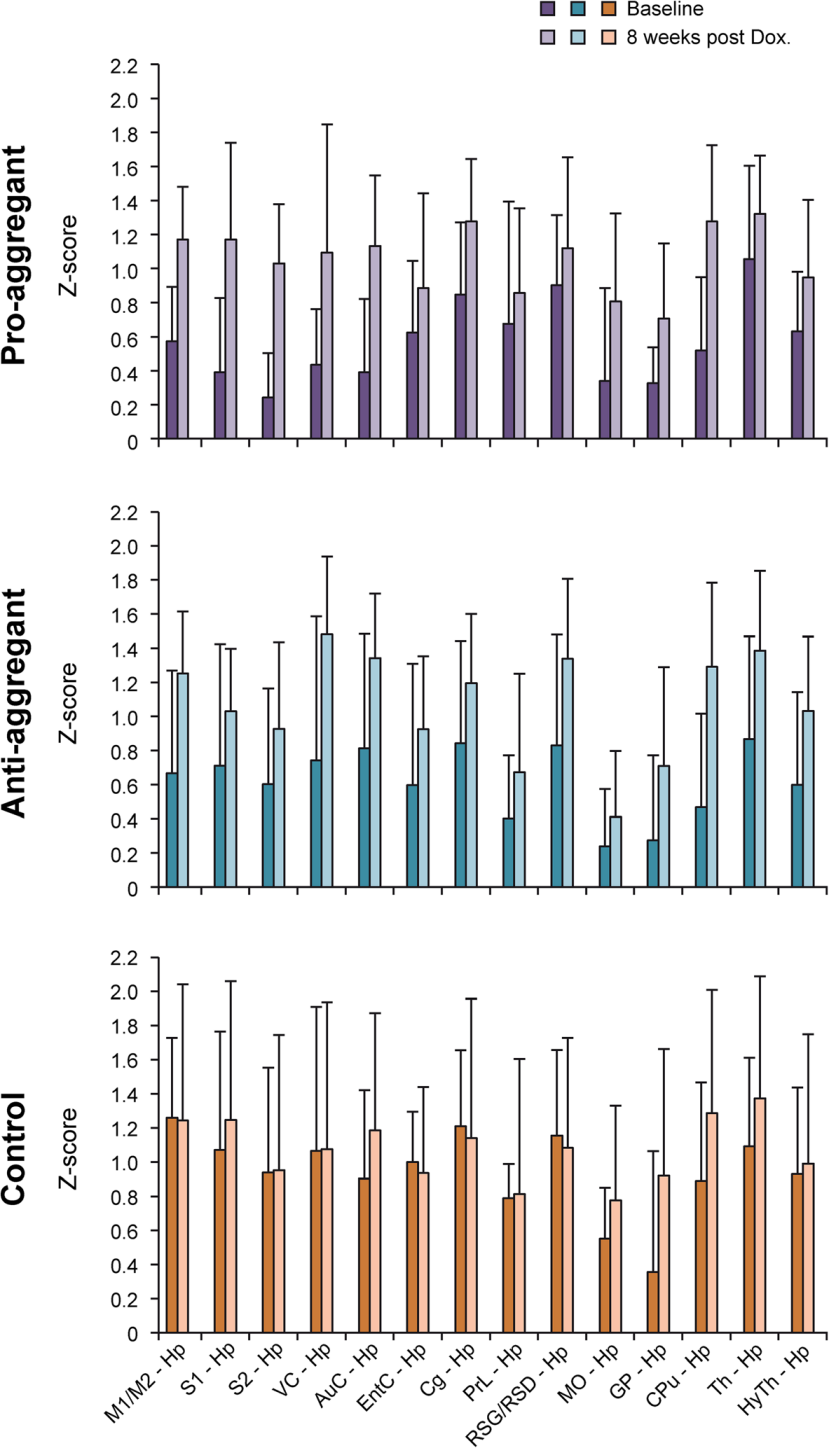
Z-score values of connections of hippocampus to cortical nodes and to nodes of the default mode network. In the control group, the changes between the two time points (baseline; doxycycline treatment for 8 weeks) remain rather small with the exception of some nodes of the DMN. In the transgenic groups, the z-score values at baseline are all only approximately half as strong as for the control group. Upon doxycycline treatment, those z-score values all closely approximate those of the control group

The hippocampus is known to be a particularly sensitive region to AD-induced alterations and one of the early regions to be affected by tau pathology. Thus, we drew particular attention to the functional changes in connectivity strength of the hippocampus with all other regions of our analysis. The treatment dependent change of these z-score values is presented in Fig. 3. In the control group, most connectivities remain unchanged upon doxycycline treatment; but an increase in connectivity of the hippocampus with nodes of the DMN is noted. In the transgenic animals, the z-score values of hippocampus to other brain regions are on average only approximately half of the comparable value in the control animals (Fig. 3). Upon doxycycline treatment, all z-score values show a clear increase reaching values similar to control animals (anti-aggregant) or only slightly below control animals (pro-aggregant).

### Regression analysis of time profile of functional connectivity matrices

**Fig. 4:**
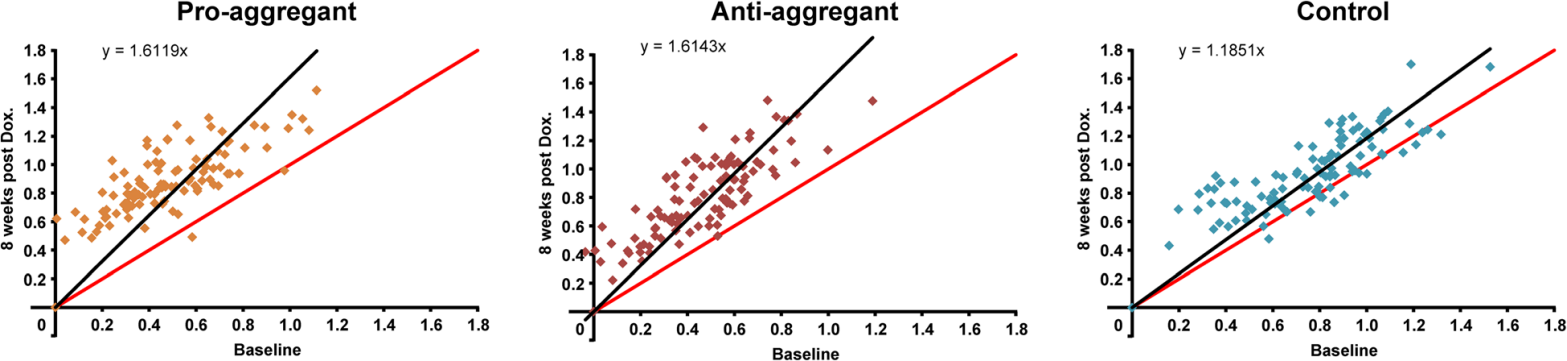
Scatter plots of matrix elements between two time points. Deviation of the scatterplots from the identity line is presented for the two time points in each diagram, for each group. The identity line is marked in red, while the fit through the scatter plot passing through zero is given in black. The slope of the fit, in deviation from slope = 1 for the identity line, indicates the overall increase or decrease of the z-score values from one time point to the other. The diagrams present the situation between baseline and doxycycline treatment for 8 weeks, showing the slope is always > 1 for all groups and indicating the z-score value increase upon doxycycline treatment (reflecting switch-off of human tau expression). This is particularly pronounced for the transgenic groups with slope of 1.612 and 1.614, respectively, demonstrating a strong overall z-score increase upon treatment, while the control group has only a slight deviation from identity with a slope of 1.18

Rather than highlighting of individual correlations between specific ROIs above, we also aimed to illustrate the overall functional differences after treatment. Scatter plots of all functional connectivity matrix elements between the two consecutive time points were generated. In the case of no changes of the individual matrix elements between two time points, all points would be expected to lie on the central diagonal through zero with slope 1 (identity line). Deviation of the fitted slope from the central diagonal (slope = 1.0, red line in Fig. 4) hereby indicates the overall increase or decrease of the z-score values from the first to the second time point for all three animal groups.

Both transgenic animal groups show a strong increase from baseline to treatment time point at 8 weeks, seen by the pronounced deviation of the fitted line from the identity diagonal (slope_pro_ = 1.612; slope_anti_ = 1.614).

In the case of the non-transgenic control group, despite a lack of statistical significance for the individual correlation values between regions, a slight increase from baseline to 8 weeks doxycycline treatment is seen via the shift of the fitted line (slope = 1.185) towards the 8 weeks treatment time point. For the control group no change over time or only an age dependent decrease should be expected. Therefore the increase at the treatment time point must be considered as due to a systemic, unspecific effect of doxycycline.

### Structural connectivity

**Fig. 5:**
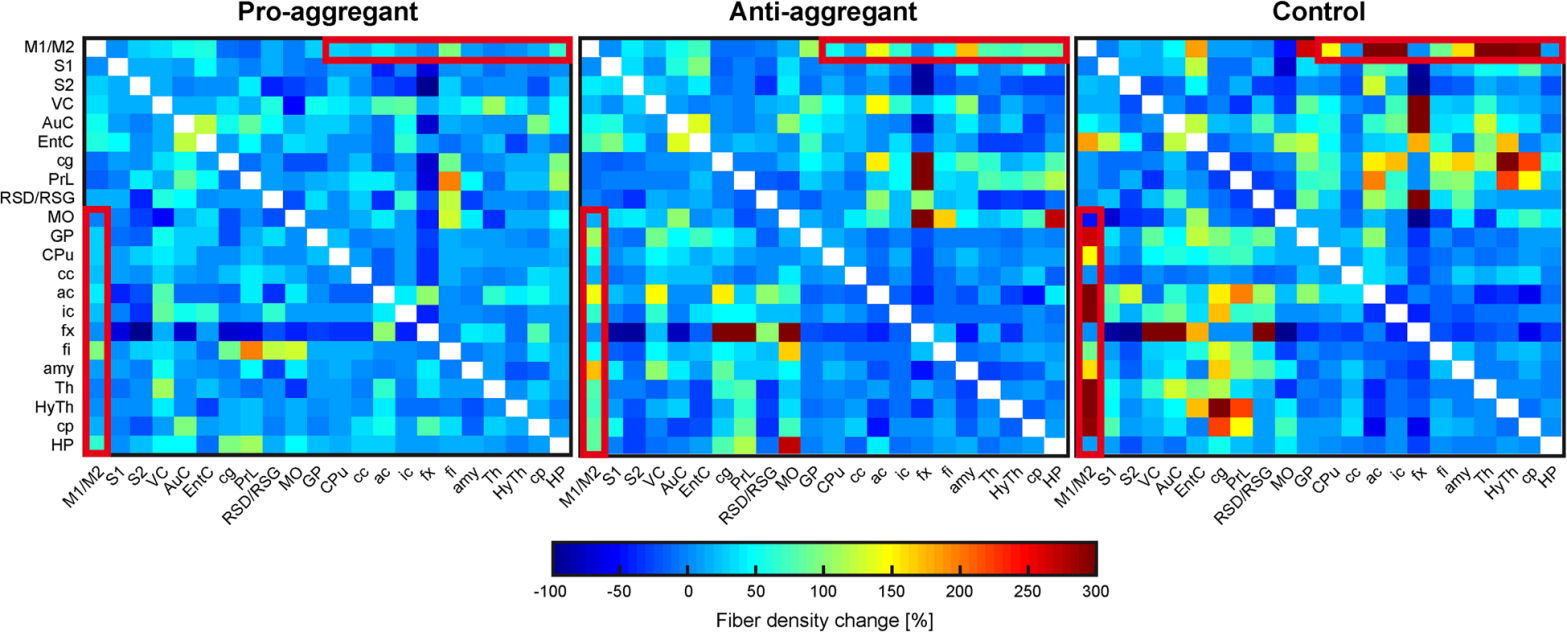
Structural connectivity matrices of fiber tracking. The matrices present the difference of fiber density between baseline and 8 weeks doxycycline treatment. Marked with a red box is the connectivity of motor cortex with various regions, particularly the white matter nodes, such as the anterior commissure, the internal capsule, and the thalamus, that also contribute to the corticospinal tract. A noticeable change of structural connectivity is also seen in the fimbria-fornix complex with most cortical nodes, contrasting a negative impact on the connections with the sensorimotor cortex and a positive increase for the connections with the cortical nodes that are associated with the default mode network

In parallel to the functional connectivity, the structural connectivity changes, obtained from fiber tracking data, were analyzed. Figure 5 presents the voxel-normalized changes in fiber density between individual nodes of the consecutive time points. A pronounced effect is seen for the connection of the motor cortex with various regions, particularly the white matter nodes, such as the anterior commissure, the internal capsule, and the thalamus, that also contribute to the corticospinal tract (CST) (red box in Fig. 5). These connections increase during the 8 weeks of doxycycline treatment, most clearly in the control group. In addition, all groups show a noticeable change of structural connectivity in the fimbria-fornix complex with most cortical nodes, contrasting a negative impact on the connections with the sensorimotor cortex and a positive increase for the connections with the cortical nodes that are associated with the default mode network.

## Discussion

In the present study, we show a definite alteration in functional connectivity, but not structural connectivity, with respect to human tau expression. This study investigates for the first time the role of tau for Alzheimer’s disease with combined structural and functional connectivity on a mouse model of tauopathy.

We report here the unexpected result that the functional brain networks respond to the expression of extra TauRD, independent of its aggregation and cognitive decline. This observation points to a novel property of tau, while previous results pointed to a role of aggregation propensity in reaching a pathological state.

The major finding in our study reveals a link between functional connectivity and overall tau expression, independent of the presence of tau aggregates. Our baseline functional connectivity measurement of the pairwise ROI correlation coefficients revealed a lower connectivity strength throughout all connections for the pro-aggregant mice compared to the wild type control group, as we had anticipated from earlier observations. Strikingly, however, we also observed an equivalent overall functional network behavior for both groups of transgenic mice at baseline measurement during full expression of human tau, i.e. before the switch-off of expression. The astonishing similar result could be clearly distinguished from the overall higher connectivity strength of the non-transgenic wildtype control group (cf. Figure 1). This situation of reduced functional network strength in both, pro- and anti-aggregant animals is one of the novel results of this study. Interestingly, the pro-aggregant mice generated full tau pathology with prominent tau aggregation and cognitive deterioration as described earlier [28], whereas the anti-aggregant mice have no tau aggregates and corresponding behavioral changes. Shifting the focus from the tau tangles to the overall presence of tau, we find a distinct relation between the amount of soluble tau, developed in both anti- and pro-aggregant models [28], and functional connectivity strength. We observe a strong increase in functional networks after switching off human tau expression in parallel with the disappearance of transgenic tauRD in both transgenic mouse models. Our observation shows a pronounced treatment effect by suppressing the expression of human tau, resulting in normalization of functional network strength, as compared to the non-transgenic wildtype mice. In consequence, already the increase of tau concentration, independent of solubility or aggregation, affects the functional neuronal networks in a subtle way that has not been observed in terms of histological changes or cognitive deficits by standard behavioral testing. The reasons of this effect are currently not known, but apparently not related to aggregation because anti-aggregant tauRD is resistant to polymerization. The cognitive deficits induced by pro-aggregant tauRD are closely correlated with impaired synapses (e.g. loss of synaptic proteins and of synapses, see above). However, tau is known to interact with many cytoplasmic cell components, including axonal microtubules, which in turn regulate axonal growth, transport, or signalling. We speculate that some of these functions are affected even by anti-aggregant tauRD which might then change functional networks in a subtle way.

Our findings are in accordance with recent studies which hypothesize that the toxic effect originates from the soluble tau (monomeric or oligomeric), promoting trans-neuronal tau propagation and disease progression, rather than the tau aggregates, as the presence of NFT in neurons cannot be the sole reason for neurodegeneration and cognitive decline [3, 8, 9, 50–55]. However, up to now these findings were not validated with in vivo functional MR imaging. Kuchibhotla and colleagues performed a study on Tg4510 mice that express a human mutant tau (P301L) and develop progressive neurofibrillary tangles in the cortex with age [53]. They observed via in vivo two-photon calcium imaging that neurons with a high NFT burden were still fully functional and neuronally integrated, arguing that NFTs are not the main cause for AD. A recent immunohistopathological study of two tauopathy rat models even suggested an almost protective role of the NFT, preserving neuronal functioning and survival [51]. However, this promising study was only performed in young adult rats and needs additional exploration. A further study on the Tg4510 mouse model was conducted using in vivo intracellular and extracellular electrophysiological recordings of neocortical pyramidal cells in mice with advanced disease pathology [54]. Their measurements on single neuron activity indicate, in line with our present results, that already an increased tau concentration affects the functional activity recordings and thereby even reduces the activity of downstream neurons, still considered unaffected. These findings were considered unlikely to arise exclusively from fibrillary forms of tau. Some studies also focused on studying the isolated effect of injected soluble tau, in the absence of NFT and discovered severe neuronal loss, as well as subsequent cognitive deficits [56]. Thus, our present study suggests that functional connectivity could become a future novel diagnostic marker of tauopathies to detect disease onset even earlier than detectable amounts of tau tangle or aggregation formation.

Furthermore, the analysis of the non-transgenic littermates demonstrated a non-uniform behavior as well, since functional connectivity increased for some somatosensory ROIs 8 weeks after onset of doxycycline feeding. Possible reasons for the altered connectivity strength, independent of pathological events which will be discussed next.

A confounding factor that could have a potential impact on neuronal signaling is the long-term administration of doxycycline. Although doxycycline is commonly used in preclinical mouse models to switch states of selected gene expressions, no investigations on the sole effect of the long-term uptake on the cerebral system of this antibiotic have been described yet. We specifically included wildtype mice in the treatment to obtain a measure of the effect of the antibiotic itself. A human study on the effect of the gut-specific broad-spectrum antibiotic rifaximin has shown an increase in functional connectivity after administration [57]**.** Its interpretation for our findings should be taken with caution, since the study was not performed on healthy subjects, yet it fits well with our own recent observations of increased functional connectivity in adult mice raised completely germ-free compared to animals in normal, specific-pathogen-free housing (unpublished results). Thus, our observed slight increase of functional network strength may in part be due to a decrease of the gut microbiome by the high dose chronic antibiotic doxycycline treatment. This highlights the need for investigating the consequences on functional connectivity by long-term antibiotic administration. The present study shows the first observation of possible systemic effects by a long-term administration of doxycycline leading to slight strengthening of the functional network on the control group.

In contrast to the results on functional connectivity, the structural connectivity was only minimally affected before and throughout treatment. On the one hand this does not seem too surprising, as significant changes in fiber tracking depend on a substantial loss of neuronal pathways. Furthermore, fiber tracking does not directly translate to a certain amount of axon bundles or axon thickness. Multiple processes might result in an altered amount of counted fibers, such as anisotropy and changes in extra- as well as intracellular space. We detected an increase in fiber density for the motor cortices with most subcortical ROIs, as well as for the fornix and most cortical ROIs of the anti-aggregant mice and the wildtype control group, between the baseline measurement and the measurement after eight weeks of doxycycline treatment. This observed strong increase cannot be reliably explained merely by the increase in structural pathways, but could originate from bystander effects in an unknown fashion. Further advanced imaging methods, such as NODDI [58], may be able to discriminate changes between the extra- and intracellular space and could thus help to identify possible causes in future studies. In any case, our results indicate that the gross structural white matter architecture appears to be preserved and largely intact, regardless of clearly noticeable changes on the functional side.

## Conclusions

Although the presence of tau aggregates is a distinct hallmark of Alzheimer’s disease, it is further accompanied by a strong accumulation and aggregation of the amyloid beta (Aβ) protein to Aβ plaques. The key drivers of the disease and their interplay are still a matter of discussion. The role of Aβ and of Aβ plaques has been a focus of preclinical animal studies. In our research, we aimed to characterize the influence of tau and its tangles in defined pathological conditions to assess specific brain-wide functional and structural pathological changes. Our findings thus cannot be directly transferred and compared to studies performed in human Alzheimer patients, experiencing full pathophysiological events, but provide important directions for further studies in this complex disease.

Taken together, our in vivo investigation unravels for the first time a strong reduction of functional neuronal networks by the presence of increased soluble rather than fibrillary tau, independent of its intrinsic propensity of aggregation, which is reversible by switching the toxic tau off. Our functional MRI study thus is the first in vivo validation of earlier reports that the increased level of soluble tau produces a toxic effect. Our results present further evidence for early tauopathy biomarkers or a potential early stage drug target.

From our study new points emerge that motivate further investigations. It would be of great interest to validate our results in a younger cohort of mice, where tau expression could be switched on again at a later stage in the study after its earlier suppression. Additionally, mouse models expressing different mutants of tau that allow investigation of synaptic neuronal pulse facilitation, as described in [59, 60], are of high interest for further studies for their effects on functional neuronal network alterations.

## Additional files

Additional file 1: **Figure S1.** Western blot results of tau expression in mice expressing pro- or anti-aggregant human tau repeat domain before and after switch-off. Lanes 1, 3: Pro-aggregant or anti-aggregant TauRD was expressed for ~ 12 months (ON), then analyzed by Western blotting. Lanes 2, 4: Pro- or anti-aggregant TauRD was expressed for ~ 12 months (ON), then switched off by doxycycline for 2 months (OFF). Hippocampal brain tissue was subjected to Western blotting for tau protein levels using antibody K9JA. In the absence of doxycycline (Tau-ON), the Western blots show human Tau^RD^ (Mr~ 14 kDa, pro-aggregant ON and anti-aggregant ON) and full-length mouse Tau (Mr~ 55 kDa) (lanes 1,3). After expression of pro- or anti-aggregant TauRD and then switching-off, the band of human Tau^RD^ has disappeared (lanes 2,4). Wildtype mice show only full-length mouse Tau (lane 5). (TIF 969 kb)

Additional file 2: **Figure S2.** Statistical analysis of functional connectivity difference between two time points. The matrices present the nodes with statistically significant difference between baseline and 8 weeks of doxycycline treatment. Only the results for the transgenic animal groups are shown as only two connections (cingulate cortex with prelimbic cortex (*p* = 0.0498) and visual cortex with globus pallidus (*p* = 0.0498)) were significantly different in the control group. (TIF 535 kb)

## Abbreviations

ac: Anterior commissure
AC: Auditory Cortex
AD: Alzheimer’s Disease
amy: Amygdala
BOLD: Blood oxygenation level dependence
cc: Corpus callosum
Cg: Cingulate cortex
cp: Cerebral peduncle
CPu: Caudate putamen
CST: Corticospinal tract
DMN: Default mode network
dMRI: Diffusion-MRI
EntC: Entorhinal cortex
FC: Functional connectivity
fi: Fimbria
FOV: Field of view
FTD: Frontotemporal dementia
fx: Fornix
GE-EPI: Gradient-echo echo-planar-imaging
GP: Globus Pallidus
Hp: Hippocampus
HyTh: Hypothalamus
ic: Internal capsule
LUT: Look-up Table
M1/M2: Primary and secondary motor cortex
MO: Medial orbital
MRI: Magnetic resonance imaging
NFT: Neurofibrillary tangles
NODDI: Neurite orientation dispersion and density imaging
PHF: Paired helical filaments
PrL: Prelimbic cortex
PSP: Progressive supranuclear palsy
QBI: Q-ball imaging
RARE: Rapid acquisition with refocused echoes
ROI: Region of interest
RSD/RSG: Retrosplenial dysgranular/granular cortex
rs-fMRI: Resting state functional MRI
S1: Primary somatosensory cortex
S2: Secondary somatosensory cortex
SE-EPI: Spin-echo echo-planar-imaging
TauRD: truncated tau repeat domain
TE: Time of echo
Th: Thalamus
TR: Time of recovery
VC: Visual cortex
