# Supplementary figures and images for: Functional networks are impaired by elevated tau-protein but reversible in a regulatable Alzheimer’s disease mouse model
Source: Mol Neurodegener. 2019 Mar 27;14:13. doi: 10.1186/s13024-019-0316-6 (PMC6438042; doi:10.1186/s13024-019-0316-6)

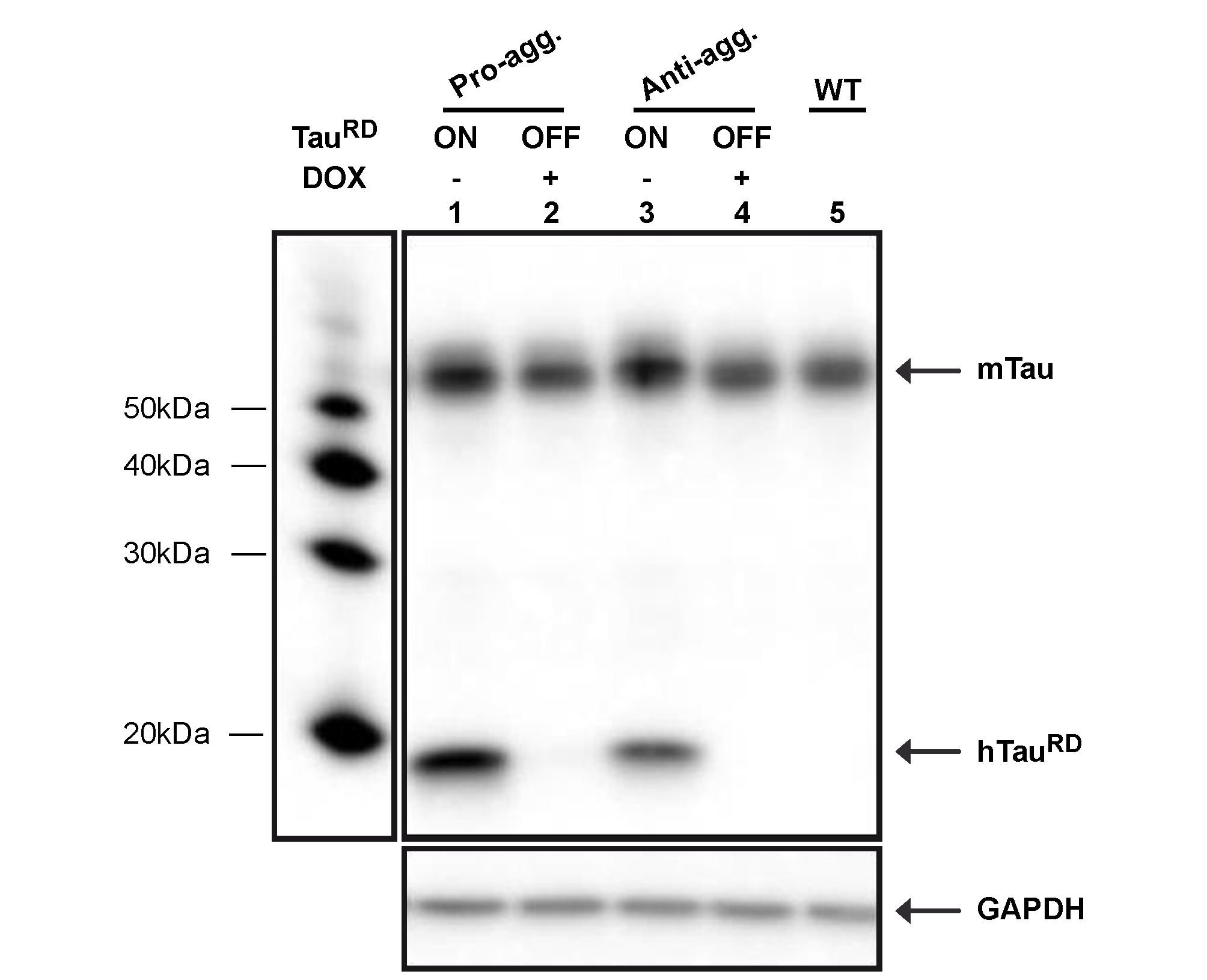

Supplement: Supplementary file 1 — Figure S1. Western blot results of tau expression in mice expressing pro- or anti-aggregant human tau repeat domain before and after switch-off. Lanes 1, 3: Pro-aggregant or anti-aggregant TauRD was expressed for ~ 12 months (ON), then analyzed by Western blotting. Lanes 2, 4: Pro- or anti-aggregant TauRD was expressed for ~ 12 months (ON), then switched off by doxycycline for 2 months (OFF). Hippocampal brain tissue was subjected to Western blotting for tau protein levels using antibody K9JA. In the absence of doxycycline (Tau-ON), the Western blots show human TauRD (Mr~ 14 kDa, pro-aggregant ON and anti-aggregant ON) and full-length mouse Tau (Mr~ 55 kDa) (lanes 1,3). After expression of pro- or anti-aggregant TauRD and then switching-off, the band of human TauRD has disappeared (lanes 2,4). Wildtype mice show only full-length mouse Tau (lane 5). (TIF 969 kb) [file 13024_2019_316_MOESM1_ESM.tif]

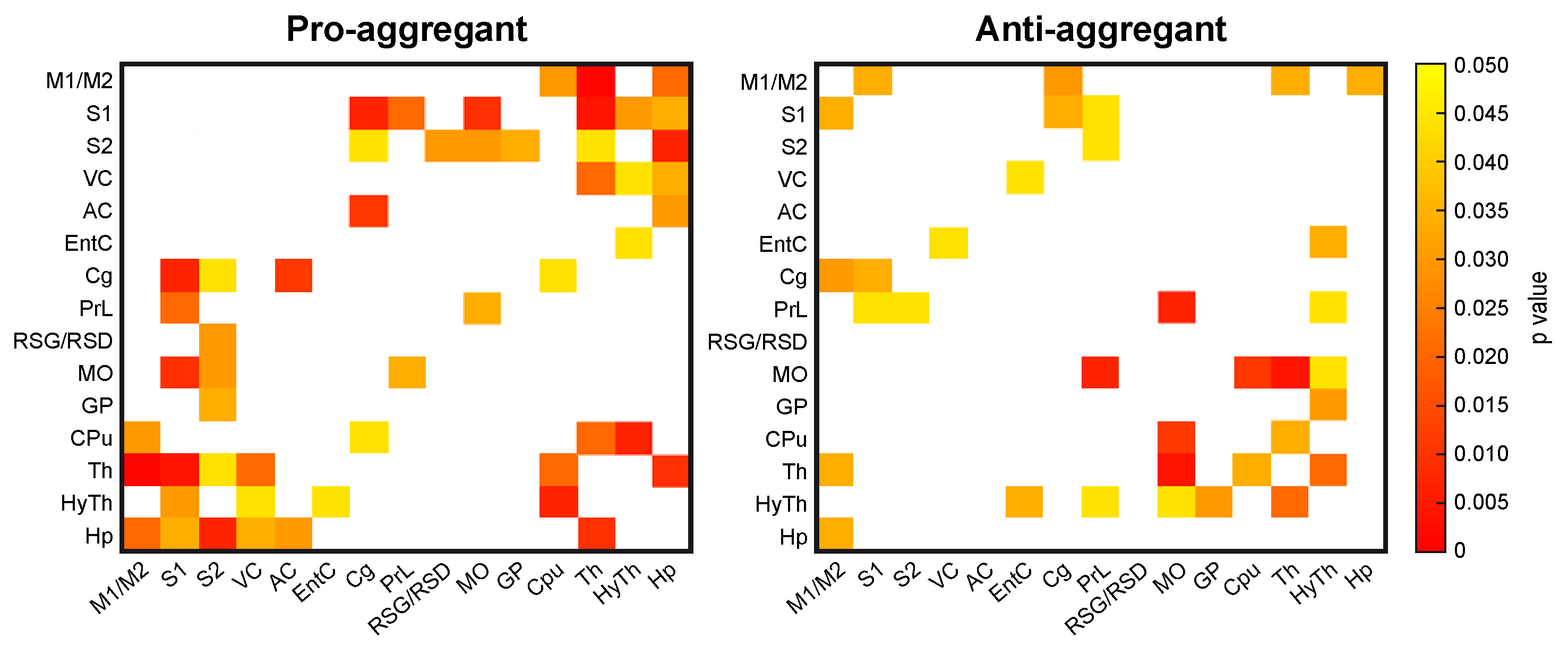

Supplement: Supplementary file 2 — Figure S2. Statistical analysis of functional connectivity difference between two time points. The matrices present the nodes with statistically significant difference between baseline and 8 weeks of doxycycline treatment. Only the results for the transgenic animal groups are shown as only two connections (cingulate cortex with prelimbic cortex (p = 0.0498) and visual cortex with globus pallidus (p = 0.0498)) were significantly different in the control group. (TIF 535 kb) [file 13024_2019_316_MOESM2_ESM.tif]
